# Clinical outcome of laparoscopic complete mesocolic excision in the treatment of right colon cancer

**DOI:** 10.1186/s12957-017-1236-y

**Published:** 2017-09-18

**Authors:** Yong Wang, Chuan Zhang, Dongsheng Zhang, Zan Fu, Yueming Sun

**Affiliations:** Department of Colorectal, the First Affiliated Hospital of Nanjing Medical University, Nanjing, Jiangsu 210029 China

**Keywords:** Complete mesocolic excision, Laparoscopy, Right colon cancer

## Abstract

**Background:**

This study aimed to investigate the clinical outcome of complete mesocolic excision (CME) with a caudal-to-cranial medial approach in the treatment of right colon cancer.

**Methods:**

The clinical data of 172 patients who underwent laparoscopic CME for right colon cancer and were admitted to the First Affiliated Hospital of Nanjing Medical University from January 2010 to April 2015 were retrospectively analyzed. The 3-year disease-free survival (DFS) and overall survival (OS) in relation to gender, age, history of abdominal surgery, tumor size, complications, and tumor–node–metastasis (TNM) classification were analyzed using the Kaplan–Meier survival curves.

**Results:**

A total of 172 patients with 94 males and 78 females were included. The average surgical time was 113.5 ± 34.4 min, blood loss was 74.2 ± 28.1 mL, and the number of lymph nodes retrieved was 23.3 ± 9.2. No readmission or death occurred within 30 days after surgery. Postoperative complications occurred in 16.3% of the patients, which included wound infection (3 patients), chylous fistula (22 patients), anastomotic leakage (1 patient), anastomotic bleeding (1 patient), and lung infection (1 patient). The 3-year DFS and OS were 81.7 and 89.1%, respectively. The rate of DFS and OS was significantly higher in stages I and II compared with that in stage III (*P* < 0.05), and in stages IIIA and IIIB compared with that in stage IIIC (*P* < 0.05).

**Conclusions:**

Laparoscopic CME with a caudal-to-cranial medial approach in the treatment of right colon cancer had good short-term efficacy and satisfactory oncological outcome.

## Background

Colorectal cancer is the most common malignancy worldwide, and surgery is the main treatment modality. Total mesorectal excision was first introduced by Heald in 1982 [1], and now it is the standard surgery for mid-low rectal cancer. In 2009, Hohenberger put forth the concept of complete mesocolic excision (CME) for colon cancer on the basis of the anatomic plane of embryonic development. By analyzing a large number of cases retrospectively, he concluded that the surgery could significantly reduce the local recurrence rate and improve the survival rate in patients with colon cancer [2]. The short-term results and long-term oncology efficacy of CME surgery were investigated in the treatment of right colon cancer through a retrospective analysis in this study.

## Methods

### Study subjects

A total of 172 patients with right colon (ileocecus, ascending colon, and hepatic flexure of colon) cancer were treated in the colorectal surgery department of the First Affiliated Hospital of Nanjing Medical University from January 2010 to April 2015. The research protocols were approved by the ethical committee of the hospital (2010-SRFA-108). The inclusion criteria were as follows: (1) preoperative pathological results indicated right colon adenocarcinoma, and the tumor was resectable; (2) no medical contraindications were observed, such as severe heart and lung diseases; (3) the patient had the ability to tolerate laparoscopic surgery including pneumoperitoneum and general anesthesia; and (4) the age range was 18–85 years. The exclusion criteria were as follows: (1) patients underwent emergency surgery due to intestinal obstruction, perforation, and so forth; (2) patients with history of other malignancies; and (3) patients with metastatic diseases.

### Surgical approach

**Fig. 1:**
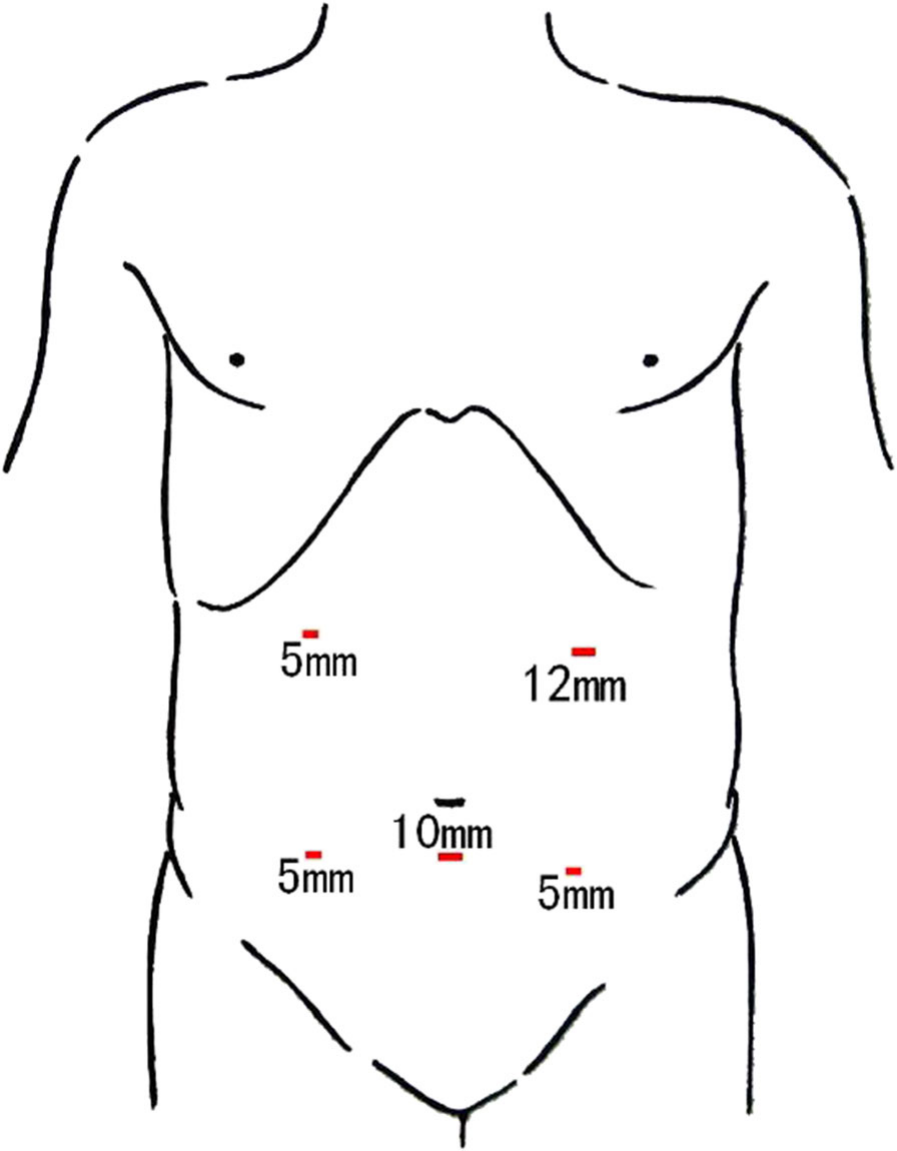
Position of operative ports

After establishing endotracheal intubation and general anesthesia, the patient was placed in a supine position. The surgeon stood on the patient’s left, the first assistant on the right, and the camera operator was located between the legs of the patient. The operative port position consisted of five sites (Fig. 1): the 10-mm camera port, placed 4 cm below the umbilicus on the midline; the 12-mm main operative port, placed at the intersection of the left mid-clavicular line and the midpoint perpendicular to the xiphoid umbilical line; two 5-mm assistant operating ports, placed at the two midpoints to the right and left of the anterior superior iliac spines and the umbilicus; and the last 5-mm assistant port, placed 3 cm below the costal margin on the right mid-clavicular line. The surgery was started using a caudal-to-cranial medial approach after routine abdominal examination.

**Fig. 2:**
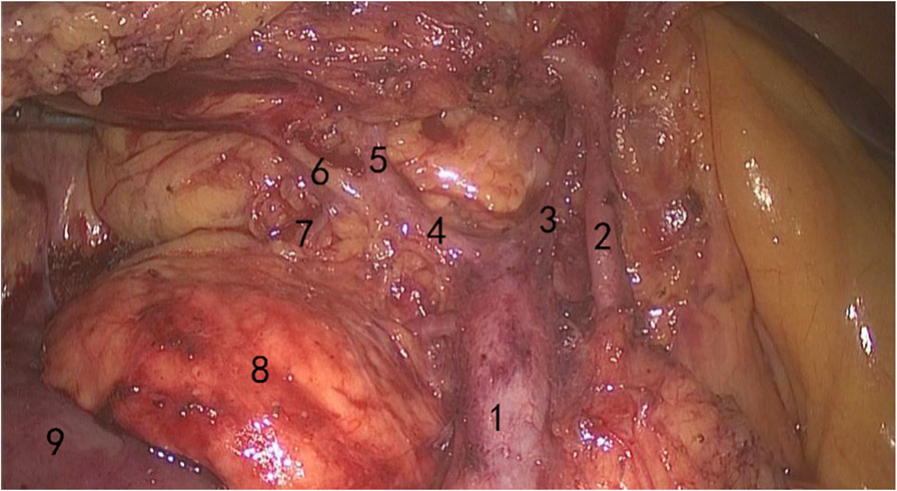
Superior mesenteric vein surgical trunk and its tributaries (*1*: surgical trunk; *2*: right tributary of arteria colica media; *3*: middle colic vein; *4*: gastrocolic trunk of Henle; *5*: venae gastroepiploica dextra; *6*: right colic vein; *7*: superior pancreaticoduodenal vein; *8*: head of pancreas; *9*: duodenum)

First, the ileocolic vessels were identified with the cecum retracted. The mesentery was opened along the ileocolic vessels to the left of the superior mesenteric artery (SMA) up to the inferior margin of the pancreas. The sheath of the superior mesenteric vein (SMV) was opened and revealed (Fig. 2). Then, oriented by the SMV, the pancreas–duodenum fascia plane was entered, and proceeding left, the lymphoid tissue along the SMA was harvested. The Toldt’s space was then entered under the ileocolic vessels and dissected anteriorly to the ligamentum hepatocolicum, outward to the lateral fusion fascia of colon, and inward to converge with the pancreas–duodenum fascia plane. After harvesting the lymphoid tissues, the ileocolic vessels were divided at the root. Following the SMV superiorly, the right colic artery (sometimes absent) was revealed and divided at the root. The two branches of the middle colic artery were dissected out. The right branch of the middle colic artery, or the middle colic artery itself in some patients, was divided at the root. The gastrocolic trunk of Henle was dissected out, the right colic vein was divided, and the venae gastroepiploica dextra was preserved after harvesting the acroteric lymph nodes (sixth group lymph nodes). The middle colic vein was divided. Proceeding from the inferior margin of the pancreas through the anterior pancreatic space, the supracolic compartment was then entered, and the gastrocolic ligament was opened and omentum majus was divided. For tumors at the hepatic flexure, the sixth group lymph nodes or nodes inside of gastroepiploic vascular arch were harvested. The ligamentum hepatocolicum was divided, and the lateral fusion fascia was converged with the dissected space below. The attachments between the ileocecus and the lateral abdominal wall were divided up to the left of the SMV to free the right colon and terminal ileum completely. A 5-cm median abdominal incision was made, and the incision was protected using a sleeve. The right colon and terminal ileum were brought out extracorporeally, and a classic right hemicolectomy was performed with a side-to-side anastomosis of the ileum and colon transversum. The bowel was then returned back into the abdominal cavity, and the incision was closed. The pneumoperitoneum was reestablished, the final inspection of the bowel was performed, and a drainage tube was placed under the liver through the right paracolic sulci.

### Perioperative and postoperative treatments

#### Preoperative preparations

Preoperative examinations were performed, which included tumor markers (carcinoembryonic antigen, carbohydrate antigen 19-9), imaging examinations [computed tomography (CT), positron emission tomography (PET)-CT], colonoscopy, and so forth; management of comorbid conditions; liquid diet and bowel prep with oral polyethylene glycol; and infusion of prophylactic antibiotics half an hour before surgery.

#### Postoperative treatments

Antibiotics and intravenous nutrition were given for 1–2 days. Liquid diet was started after intestinal function recovery 2–3 days after surgery, and transition to regular diet was started 5–7 days after surgery. Abdominal drainage tube and sutures were removed prior to discharge.

#### Postoperative adjuvant chemotherapy

Patients with stage III pathological results were given capecitabine + oxaliplatin (XELOX) or oxaliplatin + calcium folinate/fluorouracil (mFOLFOX6) as chemotherapy. Patients with high-risk stage II were given capecitabine single-agent chemotherapy. Chemotherapy was given for 6 months.

#### Evaluation indexes

The surgical time, intraoperative blood loss, number of harvested lymph nodes, time to bowel function recovery, postoperative length of stay, complications, readmission rate and mortality within 30 days after surgery, TNM stage, and postoperative tumor markers were all recorded.

### Follow-up

All the patients were served regular hospital consulting and telephone follow-up after surgery. Their abdominal B ultrasound, carcinoembryonic antigen every 3 months in the first 2 years postoperatively, colonoscopy every year, along with the chest and abdominal CT and PET-CT, if necessary, were checked. Thereafter, their abdominal B ultrasound and carcinoembryonic antigen every 6 months, 2–5 years after surgery, were checked. The follow-up began in March 2010 and ended in September 2015. Disease-free survival (DFS) meant no local recurrence or metastasis.

### Statistical analysis

The SPSS17.0 statistical software (SPSS Inc., IL, USA) was used for data analysis. The DFS and overall survival (OS) were analyzed using the Kaplan–Meier survival curves. The log-rank test was used to compare the survival rate of the two groups. A *P* value <0.05 was considered as statistically significant.

## Results

Of all the 172 patients, 94 were males and 78 were females. The age ranged from 41 to 79 (average 67 ± 12) years. Successful laparoscopic CME surgery for right colon cancer was performed in all patients. No case was converted into open laparotomy.

### Surgical data

All patients received R0 resection without ureter, duodenum, or SMV injury. The average length of surgical time was 113.5 ± 34.4 min, and the average blood loss volume was 74.2 ± 28.1 mL.

### Postoperative pathological examination

Of the 172 cases included, 10 cases were with stage I, 57 cases with stage II, 11 cases with stage IIIA, 68 cases with stage IIIB, and 26 cases with stage IIIC, based on the 2015 National Comprehensive Cancer Network (NCCN) guidelines. The histological types of the cases included were as follows: 27 cases of highly differentiated adenocarcinoma, 91 cases of moderately differentiated adenocarcinoma, and 54 cases of poorly differentiated adenocarcinoma and mucinous carcinoma. The average length of the resected bowel was 28.7 ± 5.6 cm, and the average number of harvested lymph nodes was 23.3 ± 9.2.

### Postoperative recovery and complications

No readmission and no postoperative deaths were observed within 30 days. The mean time to bowel function recovery was 2.7 ± 1.2 days; the mean postoperative length of stay was 8.7 ± 2.1 days; the short-term complication rate was 16.3% (28/172), including wound infection (3 patients), chylous fistula (22 patients), anastomotic leakage (1 patient), anastomotic bleeding (1 patient), and lung infection (1 patient). All complications appeared during the initial hospital stay and were treated during that stay. Patients with chylous fistula were treated with conservative therapy such as fasting or liquid diet without fat. Patients with anastomotic leak were treated with peritoneal lavage, nutritional support, and so on. Patients with anastomotic bleeding were treated with laparotomy and ligation of bleeding.

### Long-term oncological outcomes

The follow-up time ranged from 5 to 60 months (median follow-up time was 35 months); 31 patients failed to follow-up at different times after surgery. The successful follow-up ratio was 82.0%. During the follow-up process, 3 patients had a recurrence in the peritoneal cavity and 14 patients had distant metastasis. Of the 17 patients who died, 13 died from colon cancer and 4 from other reasons. The 3-year DFS and OS in this study were 81.7 and 89.1%, respectively.

A single-factor analysis for age, gender, history of abdominal surgery, tumor size, complications, and other clinical features with OS was performed. The results had no statistical significance (Table 1).

**Table 1:** Single-factor analysis of clinical features with 3-year OS

Factors	*n*	3-year OS (%)	*X* ^2^	*P* value
Age				
≤65	81	88.7	0.81	0.3694
>65	91	89.7		
Gender				
Male	94	88.5	0.01	0.9209
Female	78	89.7		
Abdominal surgery history				
Yes	38	95.5	0.02	0.8822
No	134	87.4		
Tumor size (cm)				
<4	41	93.4	3.17	0.2046
4–6	96	91.5		
>6	35	76.2		
Complications				
Yes	28	81.2	1.2	0.274
No	144	90.5		

*OS* overall survival

### Influences of different TNM stages on the prognosis

**Fig. 3:**
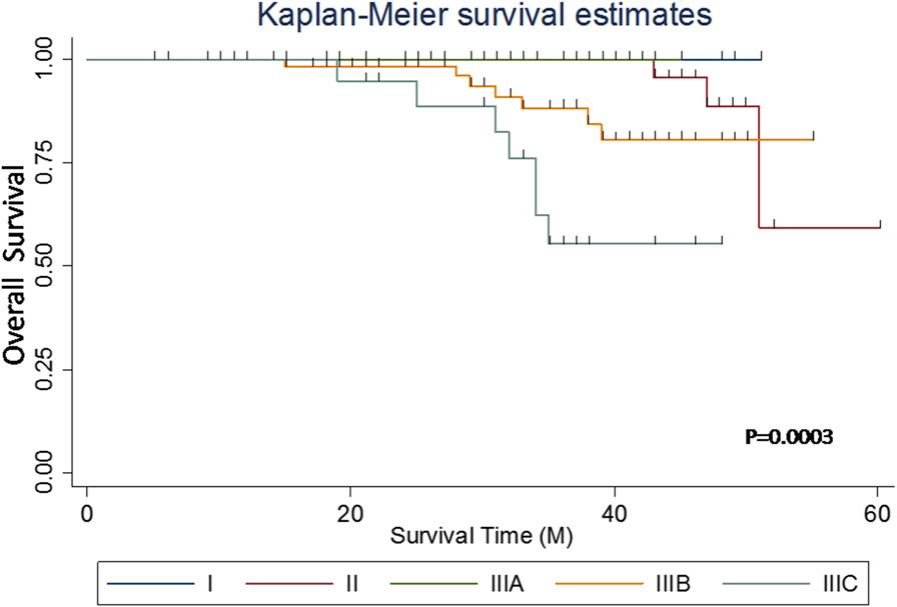
OS curves of different TNM stages

**Fig. 4:**
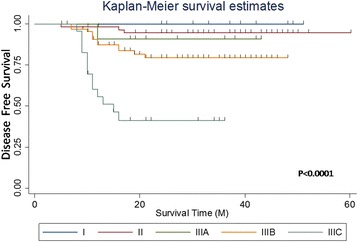
DFS curves of different TNM stages

The difference in the survival rate of the different TNM stages had statistical significance: the later the stage, the worse the prognosis. The stage IIIC patients had a much worse prognosis among the patients in stage III (Tables 2 and 3; Figs. 3 and 4).

**Table 2:** Single-factor analysis of TNM stages with prognosis

TNM stage	*n*	3-year DFS (%)	*X* ^2^	*P* value	3-year OS (%)	*X* ^2^	*P* value
I	10	100	14.16	0.0008	100	8.23	0.0164
II	57	94.6			100		
III	105	71.8			80.8		

*DFS* disease-free survival, *OS* overall survival, *TNM* tumor–node–metastasis

**Table 3:** Single-factor analysis of stage III patient prognosis

TNM stage	*n*	3-year DFS (%)	*X* ^2^	*P* value	3-year OS (%)	*X* ^2^	*P* value
IIIA	11	90.9	19.06	0.0001	100	7.27	0.0264
IIIB	68	79.4			88.2		
IIIC	26	41.2			55.4		

*DFS* disease-free survival, *OS* overall survival, *TNM* tumor–node–metastasis

## Discussion

Hohenberger proposed CME as the standard surgery for colon cancer, and the three core elements were as follows: (1) the Toldt’s space should be dissected accurately, and the mesocolon should be kept unbroken; (2) blood vessels should be ligated at the root; and (3) the regional lymph nodes should be harvested aggressively. The caudal-to-cranial medial approach for CME of right colon cancer was performed in this study. The right colic artery was dissected along the SMV from the bottom up and was ligated at the root; the lymph nodes were harvested, and a meticulous dissection was performed through the right Toldt’s space; and finally, the right colon and mesocolon were resected completely. So, the Hohenberger’s CME principle was followed thoroughly.

The key point at the beginning of the surgery in this study was to explore the fat connective tissues on the surface of the SMV and SMA adequately. The ileocolic vessels were dissected and ligated at the root. The Toldt’s space was entered accurately. The difficulty of the surgery was in identifying the gastrocolic trunk of Henle and its tributaries, arteria colica media and intermediate vein, because these vessels have many anatomic variations [3]. The surgeon should master the anatomy very well and dissect carefully to avoid damaging the vessels because bleeding from these areas would be difficult to control and might even lead to conversion to laparotomy.

The surgical time, intraoperative blood loss, number of harvested lymph nodes, time to bowel function recovery, length of stay, and complications are important benchmarks to evaluate the quality of surgery. Previous studies [4–6] reported that laparoscopic CME surgery was safe and feasible; it had good short-term results including faster postoperative recovery, shorter hospitalization time, fewer complications, and other advantages. Some studies in China [7–9] reported that laparoscopic CME for right colon cancer could improve the quality of surgical specimens and the number of lymph nodes harvested, reduce the operative blood loss, and shorten the surgical time and length of stay. The results of the present study were consistent with these reports, but the length of surgical time (113.5 ± 34.4 min) was shorter compared with previous similar reports. The number of dissected lymph nodes (23.3 ± 9.2) met the requirements of the NCCN guidelines. The length of stay was 8.7 ± 2.1 days, and the primary shot-term complication was chylous fistula (12.8%), which was closely related to the extent of lymphadenectomy. All the patients with chylous fistula were cured through conservative therapy including fasting or diet without fat for 3–5 days. Only one patient experienced anastomotic bleeding and had to be reoperated. There are several different approaches for right hemicolectomy, and medial-to-lateral approach (MA) and lateral-to-medial approach (LA) are the two main ones. The advantages and disadvantages of these two approaches are not deeply clear now, and there were not too many studies on this point either. Pingping Xu et al. [10] showed that the operation time (MA, 138.4 min vs. LA, 166.2 min; *P* < .05) and blood loss (MA, 52.0 mL vs. LA, 62.6 mL; *P* < .05) were significantly lower in the MA group. There are no statistic differences on the postoperative complications in their study. Jun et al. [11] also showed the similar results about the operative time and the estimated blood loss like Pingping Xu’s study (*P* = 0.01). There were no significant differences between the two groups in intraoperative complications, postoperative complications, number of lymph node retrieval, and hospital stay. We also compared the incidence rate of the postoperative complications between our study and these two studies and found that there were no statistic differences (*P* = 1; *P* = 0.59).

A single-factor analysis for age, gender, history of abdominal surgery, tumor size, complications, and other clinical features with a 3-year survival rate was performed in this study. The results showed that these factors had no influence on the prognosis. The 3-year DFS and OS were 81.7 and 89.1%, respectively. These were satisfactory compared with the 5-year survival rate (66%) of colorectal cancer [12], but they were slightly lower compared with those obtained in the study of Xiao Yi (DFS, 86.5%; OS, 93.7%) [13]. The influences of different TNM stages on the prognosis were also evaluated, and the survival rate was found to be significantly higher in stages I and II compared with that in stage III. This indicated that lymph node metastasis was a poor prognostic factor in right colon cancer. Similarly, the rate of DFS and OS was significantly higher in stages IIIA and IIIB compared with that in stage IIIC, and this was consistent with the follow-up results of Nakamura [14]. The 3-year OS in the present study was 100% in stage IIIA patients, which might be because the number of stage IIIA patients was too small.

## Conclusions

In summary, laparoscopic CME with a caudal-to-cranial medial approach for right colon cancer had good short-term efficacy and satisfactory oncological outcomes. Additionally, adjuvant chemotherapy is another important factor in determining the survival rate. The limitations of this study were that the chemotherapy data were not analyzed and the follow-up time was not long enough. Therefore, the patients should be followed up to validate the results of this study in the future.

## Abbreviations

CME: Complete mesocolic excision
DFS: Disease-free survival
OS: Overall survival
SMA: Superior mesenteric artery
SMV: Superior mesenteric vein
TNM: Tumor–node–metastasis
